# Structural Characterization of Polysaccharides from Partridge Tea and Their Effects on Improving FFA-Induced Lipid Accumulation in L02 Cells

**DOI:** 10.3390/foods15132273

**Published:** 2026-06-25

**Authors:** Ke-Xin Hao, Rui-Fang Zhong, Ying-Jing Zhang, Yi-Meng Li, Jian-Guo Jiang

**Affiliations:** 1 Food and Biological Engineering, Guangxi Key Laboratory of Health Care Food Science and Technology, Hezhou University, Hezhou 542899, China; haokexin2022@163.com; 2 College of Food and Bioengineering, South China University of Technology, Guangzhou 510640, China; zhongruifang2014@163.com (R.-F.Z.); yingjing_zhang@163.com (Y.-J.Z.); 3 Dermatology Hospital, Southern Medical University, Guangzhou 510091, China

**Keywords:** partridge tea, polysaccharides, structural composition, lipid accumulation

## Abstract

This study characterized the basic structure of partridge tea leaves polysaccharides and comparatively analyzed the in vitro lipid-lowering activity of total partridge tea polysaccharide (PTPS) and its two purified homogeneous fractions, namely PTPS-I (13,560 Da) and PTPS-III (30,935 Da). In terms of structural composition, PTPS-I and PTPS-III share identical monosaccharide types but differ significantly in monosaccharide proportions, glycosidic linkages and backbone structures. In vitro experiments demonstrated that PTPS, PTPS-I, and PTPS-III could effectively reduce intracellular lipid levels and oxidative stress in free fatty acids (FFA)-injured L02 cells and alleviate the decline of mitochondrial membrane potential in damaged hepatocytes. At the high concentration of 400 μg/mL, PTPS-III showed a superior effect in reducing triglyceride (TG) content compared with the other two samples, with the value reaching 0.31 ± 0.024 mmol/mg prot. Additionally, 400 μg/mL PTPS markedly decreased total cholesterol (TCHO) content and enhanced superoxide dismutase (SOD) activity, which were 0.55 ± 0.039 mmol/mg prot and 29.92 ± 0.22 μmol/mg prot, respectively. PTPS-I of 400 μg/mL significantly reduced malondialdehyde (MDA) content to 1.31 ± 0.288 μmol/mg prot and inhibited the decline of mitochondrial membrane potential (MMP) by 9.67%. The three polysaccharide fractions could elevate the mRNA expression of Nrf2, NQO1 and HO-1 in the Nrf2/HO-1 signaling pathway and the gene expression of PPARα, CPT-1 and ACOX1 in the lipid metabolism pathway, and ultimately regulate lipid accumulation in L02 cells. This study validated the in vitro antilipid activities of partridge tea leaves polysaccharide and provided fundamental data for research on its bioactivity and functional components. Further in vivo assays and mechanism exploration will be conducted to evaluate its potential application in fatty liver intervention product development.

## 1. Introduction

The liver, as an important metabolic organ in the human body, is susceptible to internal or external damage caused by drugs, immunity, alcohol, fat, and other factors, which can easily affect the body’s health [1]. The occurrence of diseases associated with non-alcoholic fatty liver disease (NAFLD), such as obesity, diabetes, and inflammation, was causing a great deal of concern [2]. Fatty liver is a disease characterized by excessive accumulation of lipids in the liver, mainly caused by the intake of large amounts of fat in the diet [3]. Excessive accumulation of triglycerides (TGs) in liver cells can cause liver damage, which in severe cases can progress from simple steatosis to non-alcoholic steatohepatitis (NASH) and even to liver fibrosis and cirrhosis or hepatocellular carcinoma [4,5]. Palmitic acid and monounsaturated oleic acid are the two highest levels of FFA in high-fat diets [6]. When FFA enters hepatocytes, it undergoes esterification to produce TG, leading to excessive accumulation of TG in the liver [7]. Mitochondria can also be damaged due to excessive accumulation of TG [8]. At present, effective treatment methods and drugs for non-alcoholic fatty liver disease need to be explored, and their safety and side effects still need further evaluation [9].

Plant-derived polysaccharides have emerged as promising hepatoprotective agents with potent lipid-regulatory activities, offering a natural and safer alternative for NAFLD intervention. For example, crude polysaccharide of *Potentilla anserina* L. markedly suppresses lipid accumulation both in oleic acid-stimulated HepG2 hepatocytes and high-fat-high-sugar induced obese mice by downregulating lipogenic genes and upregulating fatty acid oxidation-related genes [10]. Polysaccharides isolated from both fresh *Dendrobium officinale* and its processed product Fengdou can effectively reduce lipid accumulation in zebrafish models, and fresh *Dendrobium officinale* polysaccharides possess superior lipid-lowering capacity owing to their higher molecular weight, richer mannose content and more complex backbone structure [11]. Collectively, these studies fully demonstrate the broad lipid-regulating and liver-protective potential of various plant polysaccharides.

Partridge tea, scientifically known as *Mallotus peltatus* (Geiseler) Mull. arg., is a genus of wild tung in the family Euphorbiaceae [12]. It is mainly distributed in the Indochina Peninsula, Sumatra Island and Hainan Island of China; due to its dried leaves giving off a strong aroma, it is often used to make tea to drink [13]. It has been documented to have the effects of clearing away heat and detoxifying toxins, eliminating digestion and gallbladder issues, lowering blood pressure and weight loss [14,15,16]. Studies have shown that partridge tea contains a rich diversity of polyphenolic active ingredients, mainly including vitexin-4″-O-glucoside, isovitexin 2″-O-arabinoside, vitexin, spiraeoside, salicylic acid, cosmosiin, nobiletin, dimethyl phthalate and so on [13]. However, its other chemical components, such as polysaccharides, have been little studied and therefore need to be further explored.

In this study, six polysaccharides were isolated and purified from the leaves of partridge tea, of which PTPS-I and PTPS-III were used as homogeneous polysaccharides, and their structural characteristics were determined by ultraviolet (UV) and Fourier-transform infrared (FT-IR), monosaccharide composition, methylation and NMR analyses. In addition, the hepatoprotective potential of PTPS, PTPS-I and PTPS-III was evaluated in vitro using L02 cells. Their effects on the levels of antioxidant enzyme activities, MMP levels and the expression of genes closely related to the Nrf2/HO-1 and PPARα/CPT-1 pathways were assessed in FFA-induced L02 cells. These findings could lay a preliminary research foundation on the structural features and lipid-regulating bioactivity of Partridge tea polysaccharides.

## 2. Materials and Methods

### 2.1. Materials

Fresh mature leaves of Partridge tea (PT) used in this experiment were manually collected in September 2021 from Ding’an County, Hainan Province, China. Only intact and disease-free mature leaves were selected uniformly for the experiment. After collection, the leaf samples were rinsed with deionized water to eliminate surface dust, then air-dried in a cool and shaded place without direct sunlight. The dried leaves were pulverized by a high-speed plant grinder and sieved through a 40-mesh sieve to obtain homogeneous The dried powder was put into seals polyethylene bags and stored at −80 °C for reserve.

### 2.2. Extraction of Polysaccharides from PT

The powdered leaves were mixed with pure water (material/liquid ratio of 1:20 g/mL) and refluxed in an electric heating jacket (thermostat power of 800 W) for 2 h and the extraction was repeated twice. Then the supernatant was collected and concentrated to one-tenth of the original volume under reduced pressure. Subsequently, the same volume of D354FD macroporous resin was added, and placed in a magnetic stirrer at 55 °C to continue stirring for 3 h. After filtering out the resin, the polysaccharide liquid was collected and added to 4 volumes of 95% ethanol at 4 °C overnight [17]. The precipitate was dried to obtain the decolorized crude polysaccharide. Then, crude polysaccharide was re-dissolved to 5 mg/mL in water and fully mixed with 4 volumes of Sevag reagent (chloroform: n-butanol = 1:4; *V*/*V*) for deproteinization [18]. The polysaccharide solution was collected after 5 repeated deproteinizations and added with 4 volumes of 95% ethanol at 4 °C overnight. Finally, the collected precipitate was the total polysaccharide PTPS, and the yield of PTPS was calculated to be 2.16%.

### 2.3. Isolation and Purification of PTPS

The different fractions of the polysaccharide were separated using a DEAE-52 cellulose column (2.6 × 30 cm). The flow rate of the constant-flow pump was adjusted to 0.5–0.6 mL/min. A total of 10 mg/mL of PTPS was sequentially eluted using distilled water, 0.1 mol/L, 0.2 mol/L, 0.3 mol/L, and 0.4 mol/L gradient concentrations of NaCl. The polysaccharide contents were determined by phenol-sulfuric acid method [19]. The eluent obtained from each component was collected separately, concentrated under reduced pressure and dialyzed using an 8000 Da dialysis bag with running water for 48 h, then dialyzed with distilled water for 24 h. After freeze-dried, the purified polysaccharides of different components were obtained and named PTPS-I, PTPS-II, PTPS-III, PTPS-IV, PTPS-V, respectively.

### 2.4. Structural Analysis

#### 2.4.1. Physical and Chemical Property

The protein content was determined by the Coomassie brilliant blue G-250 method [20]. Briefly, different components of purified polysaccharide solution and bovine serum albumin solution were prepared to 2 mg/mL using distilled water. Moreover, 1 mL of different concentrations of standards/samples was mixed with 5 mL of Coomassie brilliant blue working solution, and the absorbance was measured at 595 nm after standing for 10 min at room temperature.

Sulfuric acid–carbazole method [21] was used to determine the content of uronic acid with glucuronic acid as the standard. Moreover, 1 mL of 0.5 mg/mL different polysaccharide samples was mixed with 5 mL of 12.5 mmol/L sodium tetraborate–sulfuric acid working solution and reacted for 20 min in a boiling water bath. Then 0.2 mL of 0.15% carbazole ethanol solution was added and remained in the boiling water bath for 5 min. The absorbance at 530 nm was measured after the reaction was carried out at room temperature for 2 h.

I-KI working solution was prepared based on the principle that starch turns blue when exposed to iodine. Moreover, 1 mg/mL of different polysaccharide samples was prepared, and 3 drops of I-KI working solution was added slowly, then left to stand for 10 min at room temperature to observe the color change [22].

#### 2.4.2. Molecular Weight and Homogeneity

Each group of purified polysaccharides was dissolved to 2 mg/mL in purified water and filtered through a 0.22 μm membrane. The molecular weight of polysaccharides was determined by gel permeation chromatography (GPC) equipped with TSK-GEL G3000_PWXL_ and TSK-GEL G5000_PWXL_ columns (7.8 mm × 300 mm), and a Waters 2414 refractive index detector (Milford, MA, USA). The mobile phase was 0.02 mol/L KH_2_PO_4_ buffer solution, with an injection volume of 20 μL and a flow rate of 0.8 mL/min. The column temperature was maintained at 35 °C [23].

#### 2.4.3. UV and FT-IR Spectroscopy

A total of 1 mg/mL PTPS-I and PTPS-III were scanned at 200–400 nm to plot the scanning curves of ultraviolet spectroscopy. Moreover, 2 mg of PTPS-I and PTPS-III were mixed with 100 mg of KBr, and the mixture was pressed into thin slices. FTIR assay was then conducted in the range of 4000–500 cm^−1^ [24].

#### 2.4.4. Scanning Electron Microscopy (SEM) Analysis

A total of 2 mg of PTPS-I and PTPS-III were taken and fixed on a metal plate using insulating adhesive, placed in a vacuum for sample gold spraying operation, and the surface morphology of the samples was scanned using a high-resolution scanning electron microscope to observe the indicated morphological variability of the polysaccharides (Carl Zeiss AG, Jena, Germany).

#### 2.4.5. Monosaccharide Composition Determination

The polysaccharide monosaccharide composition was measured by a previous reference [25]. Briefly, 10 mg of PTPS-I and PTPS-III were weighed in a hydrolysis tube and mixed with 5 mL trifluoroacetic acid (2 mol/L), then the samples were acidolysed in an oven at 110 °C for 8 h. After the samples were evaporated, 10 mg of hydroxylamine hydrochloride and 1 mL of pyridine were mixed homogeneously, added to the samples and placed in a boiling water bath for 30 min. Moreover, 1 mL of acetic anhydride was added after cool down, and the boiling water bath continued for another 30 min. Finally, the samples were filtered into gas-phase bottles using a 0.45 μm filter membrane. Moreover, 10 mg of rhamnose, fucose, arabinose, xylose, mannose, glucose and galactose were weighed as standards, and after going through the above steps, they were placed in a gas-phase vial for measurement.

Gas-phase operating conditions: Agilet 6890N gas chromatograph, FID detector; column: DB-1701 capillary column; high-purity nitrogen, flow rate 1 mL/min; heating procedure: initial temperature 180 °C, 2 °C/min heating 220 °C (maintained for 1 min), 5 °C/min heating to 250 °C (maintained for 2 min); other conditions: sample inlet temperature 250 °C, evaporation chamber temperature 250 °C, detector temperature 300 °C, flow rate 1.0 mL/min, shunt ratio 10:1.

#### 2.4.6. Methylation Analysis

Methylation analysis was referred to the previous method [26]. After 20 mg PTPS-I and PTPS-III were completely dissolved with DMSO, 200 mg of NaOH was added and shaken in a water bath at 55 °C for 6 h. In an ice-water bath environment, 5 mL of iodomethane was slowly added and sonicated for 30 min until the solution turned pale yellow, and then the reaction was terminated by adding 5 mL of water. The liquid was evaporated after dialysis with a 3500 Da dialysis bag under running water for 24 h. The above steps were repeated three times. Then the samples were extracted three times with chloroform and evaporated to dryness. After drying, the samples were added with 1 mL of pyridine and 1 mL of acetic anhydride solution and continued boiling water bath for 30 min. After three repeated washes with methanol, the sample obtained by spin-drying the liquid with a rotary evaporator was dissolved in 2 mL of CH_2_Cl_2_ and filtered into a gas phase vial using a 0.22 μm organic filtration membrane for GC-MS analysis.

GC-MS analysis conditions: Agilet 6890 N gas chromatograph, FID detector; column: TR-5MS flexible capillary column; carrier gas: high purity helium; flow rate: 1 mL/min; heating program: 150 °C (maintained for 2 min), 10 °C/min to 180 °C, 15 °C/min to 260 °C (maintained for 5 min); injection volume: 1 μL; Inlet volume: 1 μL; shunt ratio: 10:1; inlet temperature: 250 °C. Mass spectrometry conditions: transfer line temperature: 280 °C, ion source temperature: 250 °C, electron energy: 70 eV [27].

#### 2.4.7. NMR Spectroscopy

A total of 60 mg of PTPS-I and PTPS-III were weighed separately, fully dissolved in D_2_O and freeze-dried using a freeze-dryer. After repeated freezing and thawing for 3 times, the samples were dissolved in 1 mL of D_2_O, centrifuged at 4000 rpm for 5 min, and the supernatant was transferred to an NMR tube. The 1H, 13C, HSQC and COSY spectra of polysaccharides were detected by NMR spectroscopy (AVANCE III HD 600, Bruker, Billerica, MA, USA) at 600 MHz.

### 2.5. Cell Culture and Cell Viability Assay

L02 human hepatocytes were gifted by researcher Zhu Wei from the Second Clinical Hospital of Guangdong University of Traditional Chinese Medicine. L02 cells were cultured at 37 °C in an incubator with 5% CO_2_ and in DMEM (Gibco Life Technologies, Carlsbad, CA, USA) supplied with 10% fetal bovine serum (Gibco Life Technologies, USA) and 1% penicillin–streptomycin (Gibco Life Technologies, USA).

In this experiment, cell viability was determined by MTT assay [28]. Cells grown to logarithmic phase were diluted with complete medium to a density of 1 × 10^4^ cells/mL and seeded into 96-well plates at a ratio of 100 µL/well and incubated for 24 h. The culture medium was discarded and washed twice with PBS. PTPS, PTPS-I and PTPS-III were prepared in complete medium at different concentrations (50, 100, 200, 400, and 800 µg/mL) and added to 96-well plates. After 24 h of further incubation, the culture medium was discarded and washed twice with PBS. Then 100 µL of 10% MTT solution (Sigma Aldrich, St. Louis, MO, USA) was added to each well under the condition of avoiding light and incubated in the cell incubator for 4 h. Finally, the liquid was discarded, 150 µL of DMSO was added to the plate, and the absorbance at 490 nm was measured.

### 2.6. Oil Red O Staining

Referring to the methods reported in previous studies [29], 300 µM of FFA was selected as the modeling solution. Briefly, oleic acid (OA) and palmitic acid (PA) were dissolved in 0.1 M NaOH solution at 70 °C, and an equal volume of 10% bovine serum albumin (BSA) was added after mixing 1:2 by volume. After mixing well and passing through 2.5 µm filter membrane, 50 mmol/L FFA stock solution was obtained and stored at −80 °C for reserve. For the experiment, it was diluted to 300 µM with complete medium.

L02 cells in the logarithmic phase of growth were taken, and the density was adjusted to 3 × 10^5^ cells/mL with complete medium. The cells were seeded into 6-well plates at a ratio of 1.5 mL per/well and cultured for 24 h. Then the medium was aspirated and washed with PBS twice. The normal control group contained complete medium, 300 µM FFA was added to the model group and the sample groups and cultured for 24 h. After washing with PBS, normal control group and model group contained complete medium, the sample groups contained different concentration of PTPS, PTPS-I and PTPS-III (100, 200 and 400 µg/mL) and cultured for 24 h. At the end of the incubation period, the medium was aspirated, 60% isopropanol was added to cover the cells completely and stand for 20 s. Oil red O staining solution (Beyotime Biotechnology, Shanghai, China) was used for staining for 30 min. At last, the cells were washed five times with PBS and observed under the microscope (Olympus Corporation, Tokyo, Japan).

### 2.7. Determination of TCHO, TG, MDA and SOD Levels

The steps of plate laying, modeling and drug administration were carried out according to the method in Section 2.6. The cell suspension was collected and centrifuged at low speed for 10 min (1500 r/min). Then the supernatant was discarded, washed with PBS and centrifuged again. The above steps were repeated three times. The cell precipitates were broken by ultrasonication under ice-water bath conditions (power 300 W, 3–5 s/time, 30 s interval, repeated 3–5 times). The contents were determined according to the instructions of TG, TCHO kit (Nanjing Jiancheng Bioengineering Institute, Nanjing, China), MDA and SOD kit (Beyotime Biotechnology, China), and protein concentration was determined by BCA kit (Thermo Fisher Scientific Inc., Waltham, MA, USA). Each set of experiments was repeated three times.

### 2.8. Mitochondrial Membrane Potential (MMP) Assay

Cells were grouped and cultured according to the steps in Section 2.6. The cells were collected and processed according to the instructions of the Mitochondrial Membrane Potential Assay Kit with JC-1 (Beyotime Biotechnology, China). Moreover, 0.5 ml of JC-1 staining working solution was added to 0.5 mL of cells and incubated for 20 min at 37 °C. Then cells were centrifuged at 600× *g* for 3 min at 4 °C. After discarding the supernatant and washing three times with JC-1 staining buffer (1×), cells were analyzed by flow cytometry (Carl Zeiss, Oberkochen, Germany).

### 2.9. qRT-PCR Analysis

Cells were grouped for culture and administered as in 2.5. At the end of culture, the medium was discarded and the cells were washed with PBS. RNA was extracted according to the EZ-press RNA Purification Kit (EZBioscience, Roseville, MN, USA). RNA concentration was determined using a NanoDrop 2000 (Thermo Fisher Scientific, USA). OD260/OD280 between 1.90 and 2.3 was considered normal. Reverse transcription was performed according to the Color Reverse Transcription Kit instructions (EZBioscience, USA), and the PCR reaction system was configured according to the 2× Color SYBR Green qPCR Master Mix (ROX2plus) instructions (EZBioscience, USA). The amplification was performed using an ABI real-time fluorescence quantitative PCR instrument (Thermo Fisher Scientific, USA). The blank group was used as the control, and the relative expression of target gene mRNA was calculated by ΔΔCt method. Primer sequences used in RT-qPCR analysis are shown in Table S1.

### 2.10. Statistical Analysis

Data were expressed as mean ± standard deviation (SD) based on three independent biological replicates. All statistical calculations were performed using SPSS 25.0 software. Prior to one-way analysis of variance (ANOVA), the normality of data distribution and homogeneity of variances (key prerequisites for ANOVA) were validated via Shapiro–Wilk test and Levene’s test, respectively. After significant intergroup differences were identified by one-way ANOVA, pairwise comparisons between groups were conducted with two post hoc methods: the least significant difference (LSD) test and Duncan’s multiple range test. Differences at *p* < 0.05 were considered statistically significant.

## 3. Results and Discussions

### 3.1. Physicochemical Properties

Five purified polysaccharide fractions, namely PTPS-I, PTPS-II, PTPS-III, PTPS-IV and PTPS-V, were isolated from PTPS via DEAE-52 column chromatography. The standard curve equation of polysaccharide was determined as: y = 3.0591x + 0.0222, R^2^ = 0.9974. The standard curve for protein was: y = 3.4817x + 0.3038, R^2^ = 0.9951. The standard curve for uronic acid was: y = 11.696x + 0.0119, R^2^ = 0.9992. All standard curves exhibited high correlation coefficients, ensuring the accuracy of subsequent quantitative analysis. The contents of polysaccharides, proteins and uronic acids in PTPS and its five purified fractions are listed in Table 1. After the samples were placed at room temperature for 10 min, no color change was observed, indicating that starch was absent in all tested samples.

Distinct compositional differences were found among the obtained fractions, which were closely related to the adopted extraction and purification protocols. Hot water reflux extraction, macroporous resin decolorization, ethanol precipitation and repeated Sevag deproteinization were used to prepare crude polysaccharides. These treatments effectively removed pigments and most free proteins, resulting in low protein content across all fractions. Subsequent gradient elution with NaCl solution on DEAE-52 anion-exchange column achieved effective separation of polysaccharides with different charge properties. As a negatively charged component, uronic acid was predominantly enriched in PTPS-II and PTPS-III, suggesting these two fractions possessed stronger negative charge and higher acidity. Collectively, the whole extraction and purification procedures exerted a profound impact on the chemical composition and charge characteristics of the final polysaccharide fractions.

### 3.2. Molecular Weight and Homogeneity Analysis

**Figure 1:**
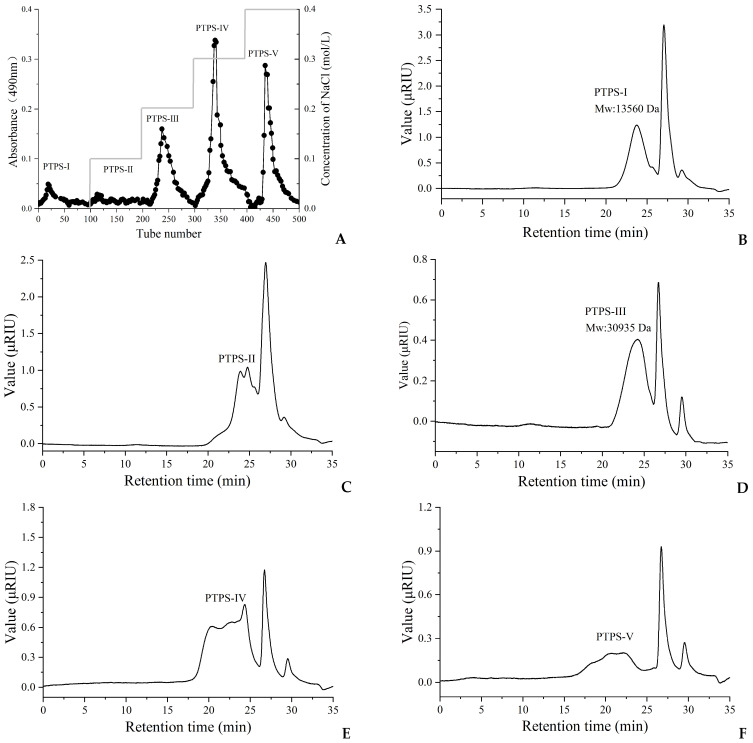
DEAE elution curve (**A**); GPC spectrum of PTPS-I (**B**), PTPS-II (**C**), PTPS-III (**D**), PTPS-IV (**E**) and PTPS-V (**F**).

PTPS was purified using a DEAE cellulose ion chromatography column, and Figure 1A showed the elution curves of each component polysaccharide. Five fractions were obtained, PTPS-III, PTPS-IV and PTPS-V had more obvious peaks.

The molecular weights and homogeneity of PTPS-I, PTPS-II, PTPS-III, PTPS-IV, and PTPS-V were determined by gel permeation chromatography (GPC). The results are shown in Figure 1B–F, in which the peaks appearing after 26 min are solvent peaks generated by the chromatographic column. The polysaccharides of each component were identified by GPC, and the elution curves are shown in Figure 1B–F. PTPS-II, PTPS-IV, and PTPS-V had heterogeneous peaks, and they could be inhomogeneous heteropolysaccharides. PTPS-I and PTPS-III possessed only one polysaccharide peak with good symmetry, and thus could be determined as homogeneous polysaccharides. According to the integration results, the Mw of the polysaccharide peak of PTPS-I was 13,560 Da, and the Mw of the polysaccharide peak of PTPS-III was 30,935 Da. Therefore, based on the above results, the structures of these two homogeneous polysaccharides were further investigated.

### 3.3. Ultraviolet and FT-IR Spectrum

**Figure 2:**
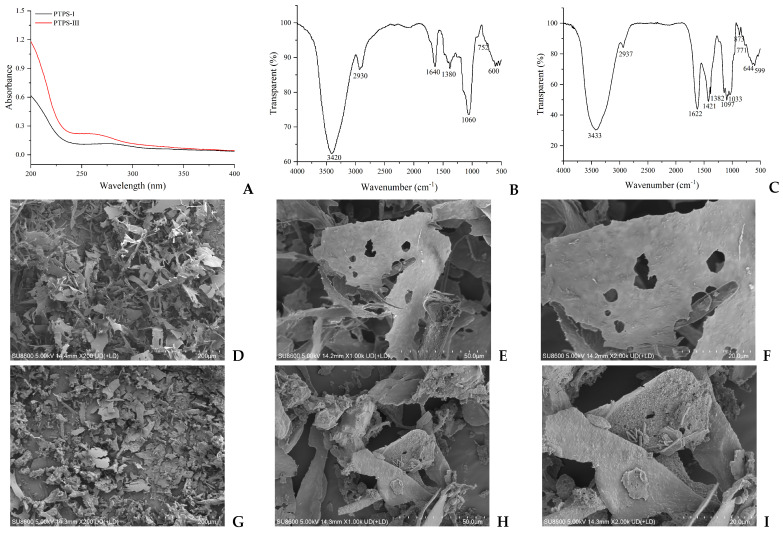
Ultraviolet full wavelength scanning diagram (**A**); FT-IR spectra of PTPS-I (**B**) and PTPS-III (**C**); SEM image of PTPS-I (**D**–**F**) and PTPS-III (**G**–**I**).

The results of full-wavelength scanning of PTPS-I and PTPS-III at 200~500 nm are shown in Figure 2A. At 260~280 nm, both PTPS-I and PTPS-III had no obvious peaks, which proved that they basically did not contain proteins and nucleic acids.

The infrared spectra of PTPS-I and PTPS-III are shown in Figure 2B,C. The absorption peaks near 3400 cm^−1^ were characteristic of the O-H stretching vibrations of the polysaccharide intermolecular bonds. The weaker absorption peaks at 2930/2937 cm^−1^ were due to the C-H stretching vibrations of the pyran ring and CH_2_ [30]. The absorption peaks at 1640 and 1622 cm^−1^ were because of asymmetric C=O stretching vibrations. The peaks appearing at 1400–1200 cm^−1^ may be C-H variable angle vibration peaks. The three overlapping peaks at 1160–1000 cm^−1^ were caused by the C-O on the sugar ring and the C-O-C stretching vibration on the glycosidic bond, which were the characteristic regional bands in the main chain of the polysaccharide glycopyranose [31,32]. The absorption peaks at 873 cm^−1^ were the equatorial bond conformations at C2 and C4 positions of the mannopyranose, and the absorption peaks at 752 cm^−1^ and 771 cm^−1^ were also indicative of a furan ring structure [33]. Moreover, 644 cm^−1^ was the CCO angular vibration peak [34].

### 3.4. SEM Analysis

Scanning electron microscopy was applied to characterize the micromorphology of solid PTPS-I and PTPS-III powder samples, and SEM images are presented in Figure 2D–I. Microscopic observation revealed that both polysaccharide powders consisted of irregular, randomly arranged sheet-like fragments. PTPS-III possessed a coarser powder surface and a denser distribution of surface pores relative to PTPS-I.

### 3.5. Monosaccharides and Methylation Analysis

**Figure 3:**
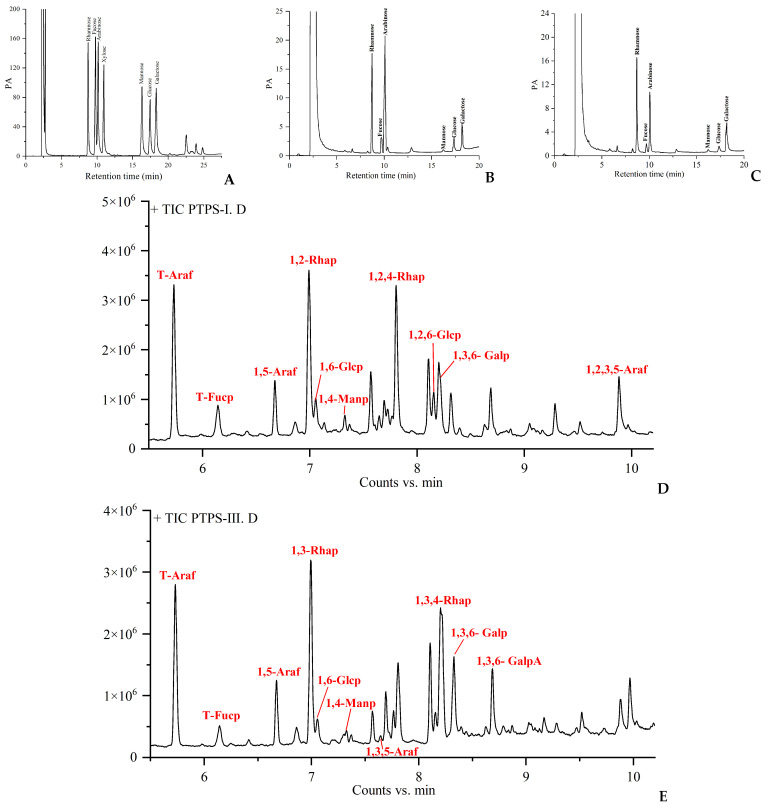
GC chromatograms of standard monosaccharides (**A**), PTPS-I (**B**) and PTPS-III (**C**); ion chromatogram from methylation analysis of PTPS-I (**D**) and PTPS-III (**E**).

As shown in Figure 3A–C, the GC results suggested that both PTPS-I and PTPS-III consisted of rhamnose, fucose, arabinose, mannose, glucose and galactose, while they had different respective percentages. Rhamnose, fucose, arabinose, mannose, glucose and galactose in PTPS-I accounted for 34.18%, 7.90%, 41.90%, 1.44%, 6.66% and 7.92%, while in PTPS-III accounted for 45.60%, 3.47%, 33.62%, 1.72%, 2.85% and 12.73%, respectively.

The specific types of sugar residues in polysaccharides need to be further analyzed by methylation experiments. Following, from Figure 3D,E and Table 2, the results of methylation analysis showed that PTPS-I consisted of T-Araf (24.76%), T-Fucp (6.22%), 1,5-Araf (6.77%), 1,2-Rhap (19.95%), 1,6-Glcp (1.63%), 1,4-Manp (1.01%), 1,2,4-Rhap (16.73%), 1,2,6-Glcp (4.46%), 1,3,6-Galp (10.93%) and 1,2,3,5-Araf (7.53%). PTPS-III consisted of T-Araf (25.30%), T-Fucp (3.71%), 1,5-Araf (7.58%), 1,3-Rhap (24.51%), 1,6-Glcp (3.32%), 1,4-Manp (1.40%), 1,3,5-Araf (0.74%), 1,3,4-Rhap (19.40%), 1,3,6-Galp (8.58%) and 1,3,6-GalpA (5.44%). PTPS-I had the highest percentage of arabinose, whereas PTPS-III had the highest percentage of rhamnose. The above results indicated that PTPS-I and PTPS-III consist of similar glycosidic bonds but with different molar ratios, implying different linkages.

### 3.6. NMR Analysis

**Figure 4:**
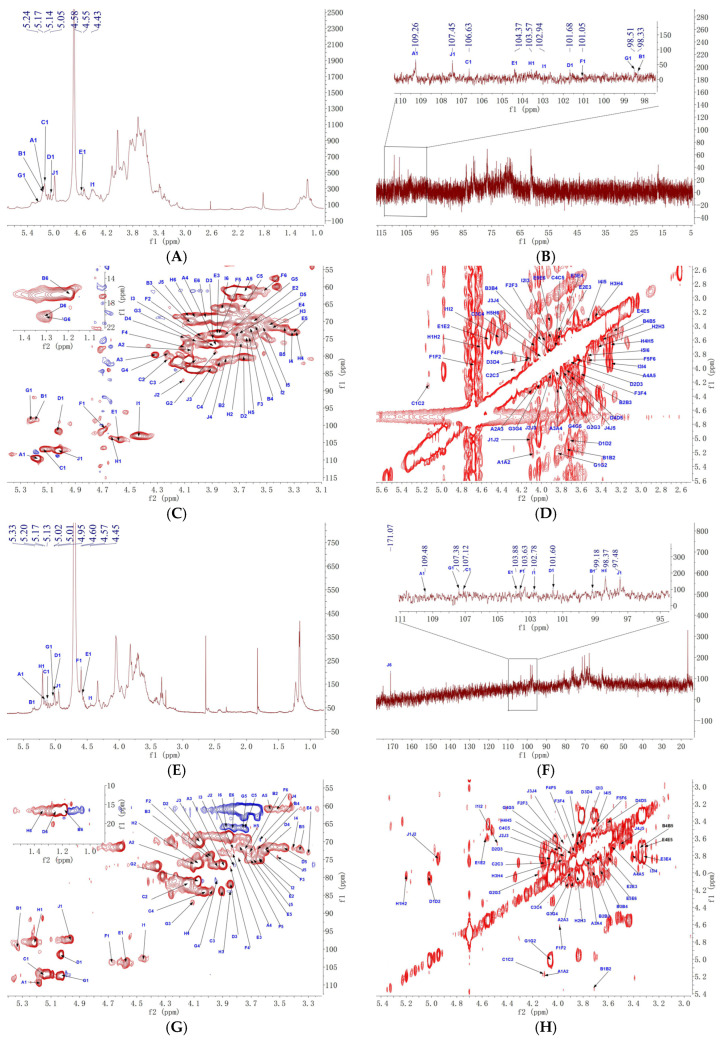
The NMR spectrum of PTPS-I: 1H spectrum (**A**), 13C spectrum (**B**), HSQC spectrum (**C**), COSY spectrum (**D**); the NMR spectrum of PTPS-III: 1H spectrum (**E**), 13C spectrum (**F**), HSQC spectrum (**G**), COSY spectrum (**H**).

The structural characterization of PTPS-I and PTPS-III was further identified by 1D and 2D NMR spectroscopy. As presented in Figure 4A, PTPS-I showed at least eight anomeric protons with overlapping signals in the anomeric region of 1H NMR at 4.36–5.12 ppm, which confirmed that PTPS-I exhibited α- and β-configurations. The corresponding anomeric carbon signals (5.17/109.26, 5.17/98.33, 5.14/106.63, 5.05/101.68, 4.58/104.37, 4.69/101.05, 5.24/98.51, 4.64/103.57, 4.43/102.94, 5.05/107.45) were identified in the 13C spectrum (Figure 4B) and HSQC spectrum (Figure 4C). Moreover, the signals at 97–105 ppm indicated the presence of pyranoside, and the signals at 106–111 ppm confirmed the presence of furanose [35,36]. Based on the methylation analysis and literature data, the signals 5.17/109.26, 5.14/106.63, 5.05/107.45 were assigned to α-Araf-(1→(A), →5)-α-L-Araf-(1→(C) and →2,3,5)-α-L-Araf-(1→(J). The signals at 5.17/98.33, 5.05/101.68, 4.58/104.37, 4.69/101.05, 5.24/98.51, 4.64/103.57, 4.43/102.94 were assigned to pyranosides, which were α-L-Fucp-(1→(B), →2)-α-L-Rhap-(1→(D), →6)-β-D-Glcp-(1→(E), →4)-β-D-Manp-(1→(F), →2,4)-α-L-Rhap-(1→(G), →2,6)-β-D-Glcp-(1→(H) and →3,6)-β-D-Glap-(1→(I). Subsequently, the H2/C2 to H6/C6 of residues were assigned according to the HSQC (Figure 4C) and 1H–1H COSY (Figure 4D) spectra. In the same way, the C/H chemical shifts in all monosaccharide residues in both PTPS-I and PTPS-III were assigned as completely as possible and listed based on the above results, 2D NMR spectra (Figure 4) and data available in the literature in Table 3 and Table 4. Collectively, both polysaccharides shared core glycosidic bond skeletons including T-Araf, T-Fucp, 1,5-Araf, 1,6-Glcp, 1,4-Manp, and 1,3,6-Galp, while their subtle differences in branched glycosidic linkages and component ratios constituted their structural discrepancies.

Structurally similar polysaccharides with identical monosaccharide compositional profiles have been widely identified in plants, and such structural features are closely associated with bioactivities of plant polysaccharides. For example, three similar pectic polysaccharides isolated from Ampelopsis grossedentata share eight identical monosaccharides but differ in molecular weight and bioactivity, and the two high-molecular-weight fractions exert stronger anti-inflammatory and antioxidant effects by inhibiting the TLR4/NF-κB pathway, confirming the close correlation between polysaccharide structure and bioactivity [37]. Six polysaccharide fractions extracted from Cordyceps militaris share similar chemical and spectral characteristics but differ in molecular weight, monosaccharide composition and anti-tumor activity, and the low-molecular-weight glucan LMW-CMP achieves potent anti-hepatoma effects via modulating MAPK/NF-κB pathways and immune functions, proving that structural variations dominate the antitumor capacity of fungal polysaccharides [38]. These studies also lay a structural foundation for the distinct effects of PTPS, PTPS-I and PTPS-III in the FFA-induced L02 cells model.

**Table 3:** Chemical shift assignments found in the NMR spectra of PTPS-I.

	Sugar Residues		Chemical Shifts (ppm)						
			1	2	3	4	5	6	References
PTPS-I	A α-Araf-(1→	H C	5.17 109.26	4.06 78.11	4.31 79.69	3.88 76.68	3.66 61.24		[39,40]
	B α-_L_-Fucp-(1→	H C	5.17 98.33	3.71 73.26	4.06 70.19	3.63 72,31	3.37 72,04	1.19 16.49	[27]
	C→5)-α-_L_-Araf-(1→	H C	5.14 106.63	4.23 81.06	4.03 81.17	3.84 81.85	3.68 65.98		[39,40]
	D→2)-α-_L_-Rhap-(1→	H C	5.05 101.68	3.67 80.23	3.90 75.02	4.10 74.63	3.70 73.19	1.30 20.26	[41]
	E→6)-β-_D_-Glcp-(1→	H C	4.58 104.37	3.60 69.87	3.86 73.53	3.48 70.87	3.31 73.07	3.15 69.03	[36,42]
	F→4)- β-_D_-Manp-(1→	H C	4.69 101.05	3.98 74.29	3.63 74.69	4.11 73.49	3.86 73.49	3.52 61.16	[39,43]
	G→2,4)-α-_L_-Rhap-(1→	H C	5.24 98.51	3.87 78.70	4.02 75.82	4.22 79.33	3.81 73.55	1.30 20.26	[41]
	H→2,6)-β-_D_-Glcp-(1→	H C	4.64 103.57	3.69 76.35	3,46 71.79	3.28 73.07	3.65 74.79	4.03 68.61	[36]
	I→3,6)-β-_D_-Galp-(1→	H C	4.43 102.94	3.56 71.47	3.89 76.64	3.31 73.14	3.53 71.82	3.83 66.49	[41,44]
	J→2,3,5)-α-_L_-Araf-(1→	H C	5.05 107.45	4.11 87.02	3.91 83.93	3.80 80.98	4.07 68.67		[39,40]

**Table 4:** Chemical shift assignments found in the NMR spectra of PTPS-III.

	Sugar Residues		Chemical Shifts (ppm)						
			1	2	3	4	5	6	References
PTPS-III	A α-Araf-(1→	H C	5.18 109.48	4.10 76.52	3.90 76.52	3.79 74.90	3.57 60.99		[39,40]
	B α-_L_-Fucp-(1→	H C	5.34 99.18	3.71 72.62	4.00 74.21	3.65 72.03	3.35 72,05	1.19 16.49	[27]
	C→5)-α-_L_-Araf-(1→	H C	5.15 107.12	4.15 81.88	3.90 83.86	4.03 84.04	3.67 61.01		[39,40]
	D→3)-α-_L_-Rhap-(1→	H C	5.01 101.60	4.06 70.02	3.85 81.98	3.60 72.39	3.47 71.64	1.25 16.72	[41]
	E→6)-β-_D_-Glcp-(1→	H C	4.57 103.88	3.65 74.90	3.83 73.63	3.30 73.14	3.70 75.16	3.83 66.44	[36]
	F→4)-β-_D_-Manp-(1→	H C	4.67 103.63	3.98 74.16	3.60 72.40	3.85 77.12	3.78 71.58	3.52 61.12	[39,43]
	G→3,5)-α-_L_-Araf-(1→	H C	5.02 107.38	4.07 101.49	4.11 86.86	3.94 80.80	3.76 66.15		[39,40]
	H→3,4)-α-_L_-Rhap-(1→	H C	5.22 98.37	4.07 75.92	3.85 83.99	3.98 83.68	3.74 72.00	1.34 16.59	[41]
	I→3,6)-β-_D_-Glap-(1→	H C	4.45 102.78	3.65 74.90	3.82 76.68	3.41 71.16	3.68 75.16	3.88 66.01	[35,41,44]
	J→3,6)-α-_D_-GlapA-(1→	H C	4.96 97.48	3.83 71.90	3.94 76.69	3.64 69.52	3.51 74.31		[41]

### 3.7. Cell Viability Analysis

**Figure 5:**
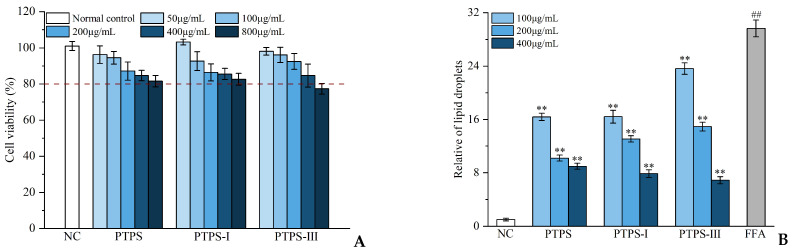

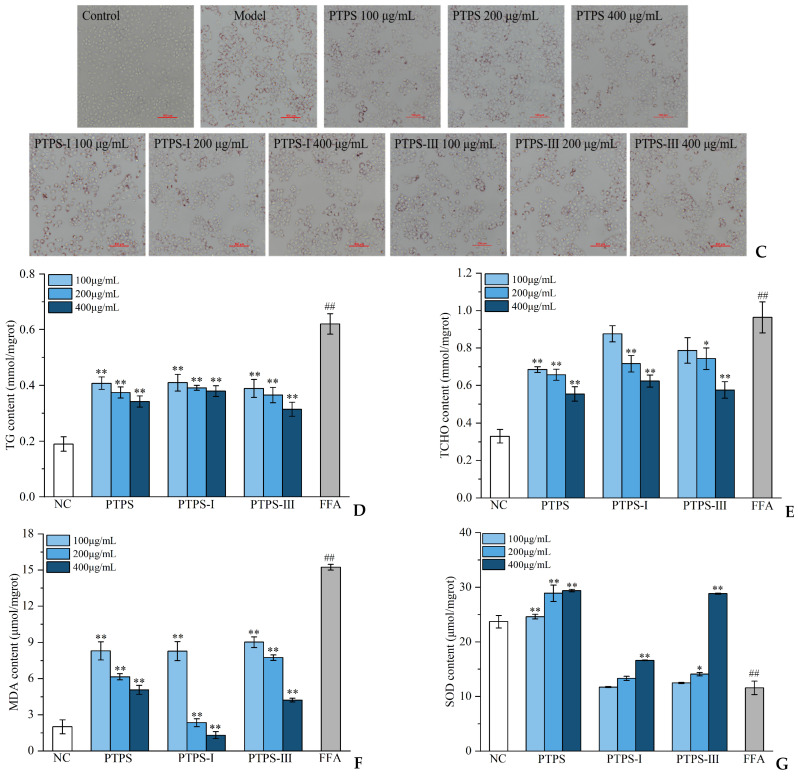
Effects of different concentrations of PTPS, PTPS-I and PTPS-III on L02 cell viability (**A**), The red dot line represents 80% cell viability; relative area of lipid droplets of PTPS, PTPS-I and PTPS-III on FFA-induced L02 cells (**B**); effects of PTPS, PTPS-I and PTPS-III on FFA-induced lipid accumulation in L02 cells (using oil red O staining) (**C**); effects of PTPS, PTPS-I and PTPS-III on TG content (**D**), TCHO content (**E**), MDA content (**F**), SOD activity (**G**) in FFA-induced L02 cells. (*) *p* < 0.05 and (**) *p* < 0.01 compared to model group, (##) *p* < 0.01 compared to control group.

The effects of the three polysaccharides on the viability of L02 cells were first detected by MTT assay. As can be seen in Figure 5A, after the three polysaccharides acted on the cells, the cell viability gradually decreased with the increase in the sample concentration, but the viability of the cells was still above 80% at the concentration of 50–400 μg/mL. When 800 μg/mL of PTPS, PTPS-I and PTPS-III acted on the cells, the cell viability was 81.55 ± 3.14%, 82.58 ± 3.26% and 77.34 ± 2.93%, respectively. PTPS-III exhibited a slight inhibitory effect. Therefore, 100, 200 and 400 μg/mL were chosen as the experimental concentrations of the three polysaccharides for the subsequent experiments.

### 3.8. Oil Red O Staining Analysis

Oil red O, as a fat-soluble dye, can serve as a colorant for neutral fats such as intracellular TG, and the larger the red area in the cells, the greater the accumulation of intracellular lipids [45]. Figure 5B,C demonstrated the effect of each group of samples on cellular lipid accumulation in L02 cells after 24 h of 300 μM FFA action. Compared with the normal group and the model group, the lipid droplets in the model group were large and numerous, while the lipid droplets were almost invisible in the normal group, indicating the success of modeling. After the three samples acted on the cells for 24 h, the lipid droplets gradually decreased with the increase in concentrations.

### 3.9. TG, TCHO, MDA and SOD Levels

The changes in the levels of TG and TCHO were illustrated in Figure 5D,E. The TG and TCHO contents in the model group were significantly increased relative to the normal group, and both TG and TCHO were decreased after PTPS, PTPS-I and PTPS-III acted on the cells. In Figure 5D, the intracellular TG contents decreased with the increase in sample concentrations. When the concentration was 400 μg/mL, the effect of PTPS-III was better than that of other samples, which was consistent with the results of Oil Red O. In Figure 5E, at a low concentration (100 μg/mL), PTPS-I and PTPS-III did not have a significant effect on intracellular TCHO contents (*p* > 0.05). After the concentration was increased, all three samples exerted the effect of reducing TCHO.

MDA is a product of lipid peroxidation, which indirectly reflects the degree of cell membrane damage [46]. As can be seen in Figure 5F, the MDA content in the model group increased significantly, while that in the sample groups decreased significantly. SOD activity is an important pathological parameter in oxidatively stressed cells, and SOD promotes the catabolism of superoxide radicals to produce hydrogen peroxide and oxygen, reducing the level of oxidative stress in vivo [47]. The effect of the samples on intracellular SOD activity was shown in Figure 5G. PTPS-I had no significant effect on increasing SOD content at 100 and 200 μg/mL concentrations (*p* > 0.05), and the SOD content at the high concentration was 16.59 ± 0.06%, which was not as effective as the other samples. The effect of PTPS-III in improving SOD activity increased with the increase in concentration, and the SOD content at 800 μg/mL concentration was 28.81 ± 0.09%, which was comparable to the effect of high concentration of PTPS (29.98 ± 0.22%). The effect of PTPS in improving SOD activity was optimal. In summary, it could be seen that all three samples can significantly ameliorate oxidative stress in high-fat L02 cells.

### 3.10. MMP Analysis

**Figure 6:**
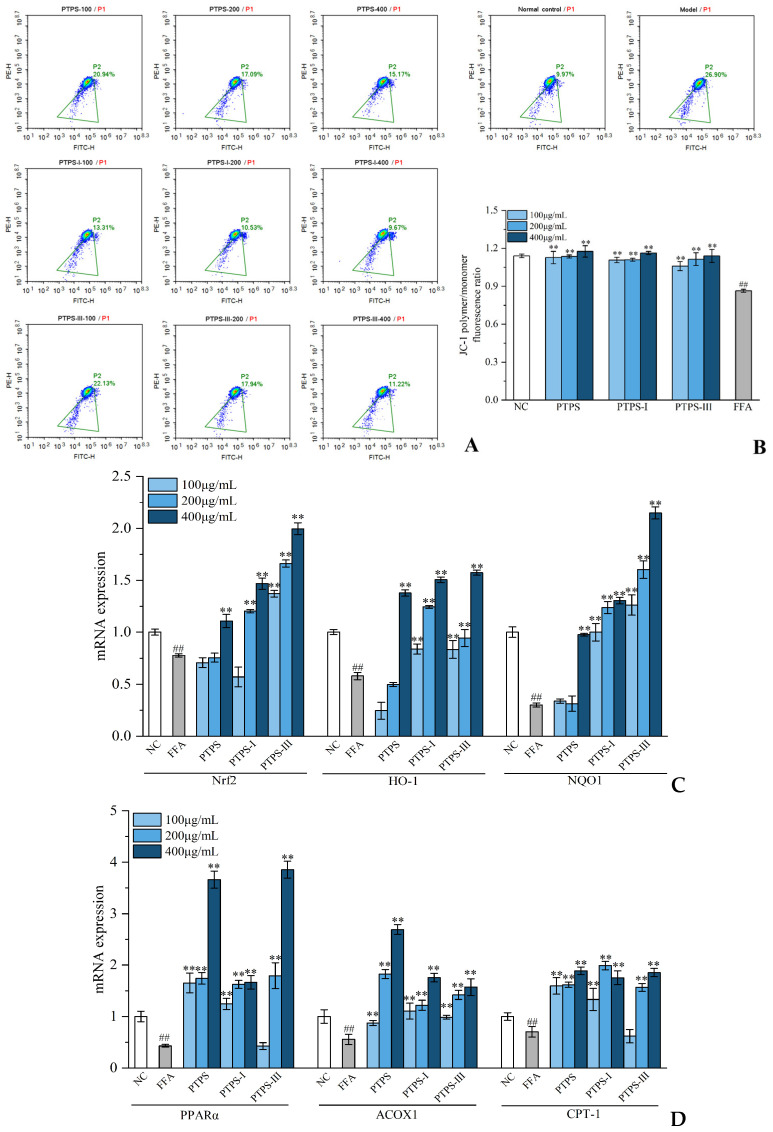
Effects of PTPS, PTPS-I and PTPS-III on MMP level on FFA-induced L02 cells (**A**) and JC-1 polymer/monomer fluorescence ratio (**B**); effects of PTPS, PTPS-I and PTPS-III on mRNA expression levels of Nrf2, HO-1, NQO1 (**C**), PPARα, ACOX1 and CPT-1 (**D**). (**) *p* < 0.01 compared to model group, (##) *p* < 0.01 compared to control group.

Mitochondria are important organelles that provide energy for cellular life activities, and MMP is the driver of ATP production and mitochondrial output [48]. The hallmark event in the early stages of apoptosis is the decrease in MMP [49]. JC-1 is a transmembrane fluorescent dye that selectively enters mitochondria and changes its fluorescent properties in response to changes in MMP. In healthy mitochondria, JC-1 usually appears as red fluorescent aggregates, while when mitochondria are damaged to some extent, MMP is reduced and JC-1 appears as green fluorescent monomers [50]. MMP levels and thus mitochondrial status can be assessed by the ratio of the number of its red/green fluorescent cell populations [51]. As shown in Figure 6A, 26.90% of the cells in the model group showed a decrease in MMP, which was 16.93% higher compared to the normal control group, indicating that FFA exerted a greater effect. As shown in Figure 6A,B, when PTPS, PTPS-I and PTPS-III acted on L02 cells, the proportion of cells with decreased MMP was significantly reduced relative to the model group, suggesting that all three were effective in ameliorating the decrease in cellular MMP.

### 3.11. Relative mRNA Expression

The occurrence of oxidative stress is the pathogenesis of obesity. Nrf2 is the link between increased adipose tissue mass and oxidative stress, and it plays an integral role in metabolic homeostasis by regulating oxidative and inflammatory responses [52,53]. HO-1 is an isoform of heme oxygenase [54] that is effective in ameliorating metabolic diseases via a lipocalin-dependent pathway [55]. The levels of genes related to the Nrf2/HO-1 pathway were shown in Figure 6C. FFA caused a significant decrease in the levels of Nrf2, HO-1 and NQO1 in cells after its action on L02 cells. Low and medium concentrations of PTPS had no significant effect on the expression of the three genes. Only 800 μg/mL of PTPS increased the transcript levels of the three genes (*p* < 0.05). With increasing concentrations, both PTPS-I and PTPS-III played the role of increasing gene levels. Overall, the effect of high concentration of PTPS-III improved the expression of Nrf2, HO-1 and NQO1 genes was significantly better than that of PTPS and PTPS-I.

PPARα is a nuclear receptor expressed predominantly in the liver that regulates the transcription of genes involved in hepatic fatty acid uptake and oxidation. Activation of PPARα increases β-oxidation of fatty acids and decreases cellular lipids [56,57]. CPT-1 is the rate-limiting enzyme for the entry of lipoyl coenzyme A into mitochondria, and ACOX1 is a key enzyme for peroxisomal β-oxidation. By up-regulating PPARα and its downstream target genes CPT-1 and ACOX1, fatty acid oxidation can be promoted and the functional integrity of mitochondria can be improved [58,59]. Figure 6D showed the effects of PTPS, PTPS-I and PTPS-III on lipid metabolism-related gene production. Under the effect of FFA, the gene expression of PPARα, ACOX1 and CPT-1 decreased to 0.43 ± 0.03, 0.55 ± 0.10 and 0.70 ± 0.10, respectively. The gene expression of PPARα, ACOX1 and CPT-1 was significantly increased by the addition of PTPS and PTPS-I at different concentrations. The low concentration of PTPS-III (100 μg/mL) had no significant effect on PPARα and CPT-1 (*p* > 0.05), but the increased concentration of PTPS-III increased the gene expression. The above results suggested that PTPS, PTPS-I and PTPS-III could inhibit fat accumulation by improving lipid metabolism.

Collectively, the above lipid-regulating experimental results demonstrated that the lipid-regulation evaluation in FFA-induced L02 hepatocyte model verified that PTPS and its purified fractions, PTPS-I and PTPS-III, exhibited favorable hypolipidemic bioactivity without significant cytotoxicity within the tested concentration range. The structural differences between PTPS-I and PTPS-III directly contributed to their varying efficacy in reducing intracellular lipid droplet accumulation, modulating oxidative stress indicators (TG, TCHO, MDA, SOD), and restoring mitochondrial membrane potential. Mechanistically, the three polysaccharides exerted lipid-metabolizing and antioxidant effects by activating the Nrf2/HO-1 signaling pathway and regulating key gene expression of the lipid metabolism pathway, demonstrating a typical structure–activity relationship.

## 4. Conclusions

This study conducted a preliminary investigation on the structural characteristics and hypolipidemic activity of polysaccharides isolated from partridge tea. Two homogeneous polysaccharides, PTPS-I and PTPS-III, were successfully purified from PTPS. Structural characterization based on monosaccharide composition and NMR spectroscopy revealed that PTPS-I and PTPS-III belong to highly branched heteropolysaccharides mainly composed of arabinose, rhamnose, galactose, and glucose residues, with low uronic acid contents. The lipid-regulation evaluation in FFA-induced L02 hepatocyte model verified that PTPS and its purified fractions, PTPS-I and PTPS-III, exerted lipid-metabolizing and antioxidant effects by activating the Nrf2/HO-1 signaling pathway and regulating key gene expression of the lipid metabolism pathway, demonstrating a typical structure–activity relationship: the unique branched chain structures of pectic polysaccharides of partridge tea are critical determinants of their hypolipidemic potency.

Collectively, this work clarifies the basic structural traits and in vitro hypolipidemic activity mechanism of partridge tea polysaccharides, providing preliminary experimental evidence for the functional development of partridge tea polysaccharides as natural lipid-regulating functional components. Nevertheless, several limitations of the present study should be acknowledged. First, this is only a preliminary in vitro investigation, and the lipid-lowering efficacy and molecular mechanism of PTPS-I and PTPS-III have not been validated via in vivo animal models, which restricts the reliability and comprehensiveness of the current findings. Second, plant polysaccharide extraction and purification processes are complicated, and the low extraction yield of partridge tea polysaccharides limits further functional exploration and application research. Third, the structure–activity relationship analysis in this study is limited to macroscopic structural differences and in vitro activity differences; the precise molecular interaction mechanism between polysaccharide structures and target proteins remains to be elucidated. Future studies will focus on optimizing the polysaccharide extraction and purification process, conducting systematic in vivo pharmacological verification, and further exploring the quantitative structure–activity relationship to supplement and improve the research system of partridge tea polysaccharides.

## Figures and Tables

**Table 1:** Percent contents of polysaccharide, protein, uronic acid and starch in PTPS-I, PTPS-II, PTPS-III, PTPS-IV and PTPS-V.

Components	Content (%)					
	PTPS	PTPS-I	PTPS-II	PTPS-III	PTPS-IV	PTPS-V
polysaccharide	72.86 ± 3.25%	96.97 ± 2.51%	82.36 ± 1.56%	85.67 ± 1.84%	82.5 ± 2.54%	84.1 ± 2.65%
protein	1.05 ± 0.07%	0.69 ± 0.22%	1.38 ± 0.20%	1.14 ± 0.12%	0.18 ± 0.06%	0.5 ± 0.10%
uronic acid	16.18 ± 1.21%	1.41 ± 0.25%	5.26 ± 1.24%	6.17 ± 0.61%	2.17 ± 0.15%	3.05 ± 0.26%
starch	-	-	-	-	-	-

**Table 2:** Methylation analysis and glycosidic linkage composition of PTPS-I and PTPS-III.

	Number	Time (min)	PMAAS	Linkage	Molar Ratio (%)	Main Fragments (*m*/*z*)
PTPS-I	A	5.731	2,3,5-Me_3_-Araf	T-Araf	26.76	58, 71, 87, 101, 117, 129, 145, 161
	B	6.145	2,3,4,6-Me_2_-Fucp	T-Fucp	6.22	43, 58, 87, 101, 115, 131, 145, 161, 175
	C	6.674	2,3-Me_2_-Araf	1,5-Araf	6.77	58, 71, 87, 99, 102, 117, 129, 159, 162, 189
	D	6.992	3,4,6-Me_3_-Rhap	1,2-Rhap	19.95	57, 71, 87, 99, 115, 130, 161, 174, 189, 207, 233
	E	7.054	2,3,4-Me_3_-Glcp	1,6-Glcp	1.63	59, 71, 87, 99, 117, 129, 143, 161, 189
	F	7.326	2,3,6-Me_3_-Manp	1,4-Manp	1.01	59, 71, 87, 102, 117, 129, 143, 173, 189, 207, 233
	G	7.805	6-Me_1_-Rhap	1,2,4-Rhap	16.73	59, 74, 87, 101, 129, 143, 159, 189, 203
	H	8.155	3,4-Me_2_-Glcp	1,2,6-Glcp	4.46	43, 71, 85, 101, 117, 129, 145, 161, 175, 207, 233
	I	8.203	2,4-Me_2_-Galp	1,3,6-Galp	10.93	58, 87, 117, 129, 139, 143, 160, 174, 185, 207, 231, 305
	J	9.880	Araf-(OAc)_5_	1,2,3,5-Araf	7.53	61, 85, 103, 115, 128, 145, 157, 175, 187, 217, 289
PTPS-III	A	5.732	2,3,5-Me_3_-Araf	T-Araf	25.30	43, 58, 71, 87, 101, 117, 129, 145, 161
	B	6.145	2,3,4,6-Me_2_-Fucp	T-Fucp	3.71	59, 71, 89, 101, 115, 117, 131, 142, 161, 175
	C	6.674	2,3-Me_2_-Araf	1,5-Araf	7.58	59, 72, 87, 88, 99, 101, 117, 129, 131, 162, 189
	D	6.993	2,4,6-Me_3_-Rhap	1,3-Rhap	24.50	59, 71, 87, 101, 117, 145, 161, 173, 205
	E	7.056	2,3,4-Me_3_-Glcp	1,6-Glcp	3.32	59, 71, 87, 99, 101, 129, 159, 173, 189, 233
	F	7.326	2,3,6-Me_3_-Manp	1,4-Manp	1.40	55, 71, 87, 101, 117, 129, 145, 161, 176, 203, 233
	G	7.643	2-Me_1_-Araf	1,3,5-Araf	0.74	85, 99, 117, 129, 145, 159, 173, 201
	H	8.21	6-Me_1_-Rhap	1,3,4-Rhap	19.40	71, 87, 99, 117, 129, 145, 157, 160, 173, 187, 202, 233
	I	8.328	2,4-Me_2_-Galp	1,3,6-Galp	8.58	73, 85, 103, 117, 129, 139, 160, 187, 217, 233
	J	8.686	2,4-Me_2_-GalpA	1,3,6-GalpA	5.44	75, 87, 100, 117, 129, 142, 159, 185, 201, 231

## Data Availability

The original contributions presented in this study are included in the article/Supplementary Materials. Further inquiries can be directed to the corresponding authors.

## Supplementary Materials

The following supporting information can be downloaded at: https://www.mdpi.com/article/10.3390/foods15132273/s1, Table S1. Primer sequences used in RT-qPCR analysis. Figure S1. Partially O-methylalditol acetates formed on methylation analysis of PTPS-I. Figure S2. Partially O-methylalditol acetates formed on methylation analysis of PTPS-III.
