# Supplementary material for: Structural Characterization of Polysaccharides from Partridge Tea and Their Effects on Improving FFA-Induced Lipid Accumulation in L02 Cells
Source: Foods. 2026 Jun 25;15(13):2273. doi: 10.3390/foods15132273 (PMC13360729; doi:10.3390/foods15132273)
Supplement: Supplementary file 1 [file foods-15-02273-s001.zip › foods-4370165-supplementary.pdf]

Table S1. Primer sequences used in RT-qPCR analysis.

| Genes | Primer sequences（5’-3’） |
|---|---|
| β-actin | Forward: CTTAGTTGCGTTACACCCTTTC<br>Reverse: ACCTTCACCGTTCCAGTTTT |
| Nrf2 | Forward: CGGTATGCAACAGGACATTG<br>Reverse: ACTGGTTGGGGTCTTCTGTG |
| HO-1 | Forward: CCAGGCAGAGAATGCTGAGT<br>Reverse: GTAGACAGGGGCGAAGACTG |
| NQO1 | Forward: CTGATCGTACTGGCTCACTC<br>Reverse: GAACAGACTCGGCAGGATAC |
| γ-GCL | Forward: GGCGATGAGGTGGAATACAT<br>Reverse: CCTGGTGTCCCTTCAATCAT |
| GPx | Forward: ATGTGTGCTGCTCGGCTA<br>Reverse: AGAAGGCATACACCGACTGG |
| SOD | Forward: GAAGGTGTGGGGAAGCATTA<br>Reverse: ACCACAAGCCAAACGACTTC |
| PPARα | Forward: TGGCTCTTGACCCTATTGG<br>Reverse: GGGAACAGATTTCCACATTG |
| CPT-1 | Forward: CCTCCGTAGCTGACTCGGTA<br>Reverse: GGAGTGACCGTGAACTGAAAG |
| ACOX1 | Forward: GCGGACTACACTTCATAAATGC<br>Reverse: CCACAGGACACCATTAAGC |
| SIRT1 | Forward: GCCTCATCTGCATTTTGATG<br>Reverse: TCTGGCATGTCCCACTATCA |

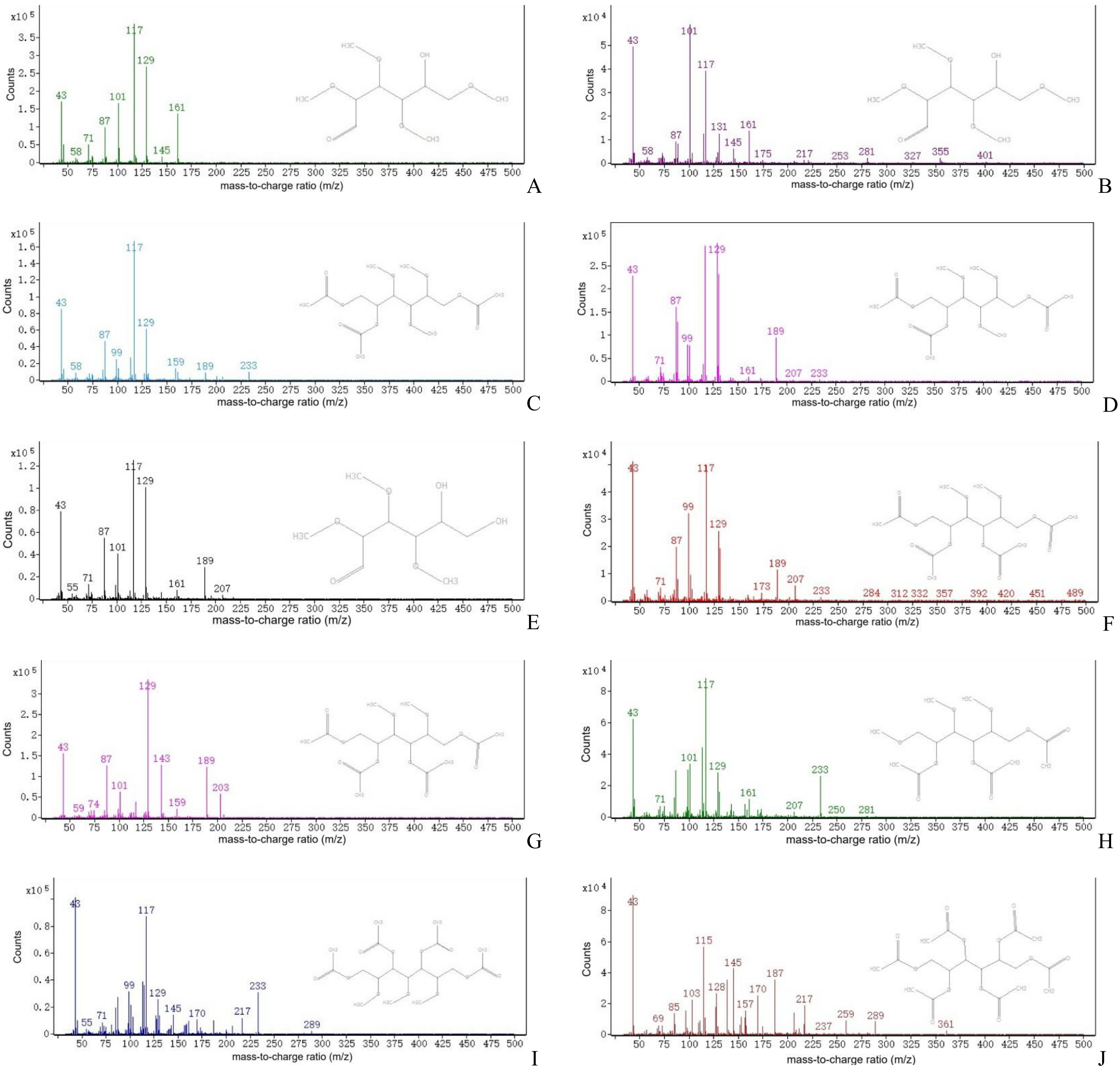


Figure S1. Partially O-methylalditol acetates formed on methylation analysis of PTPS-I.

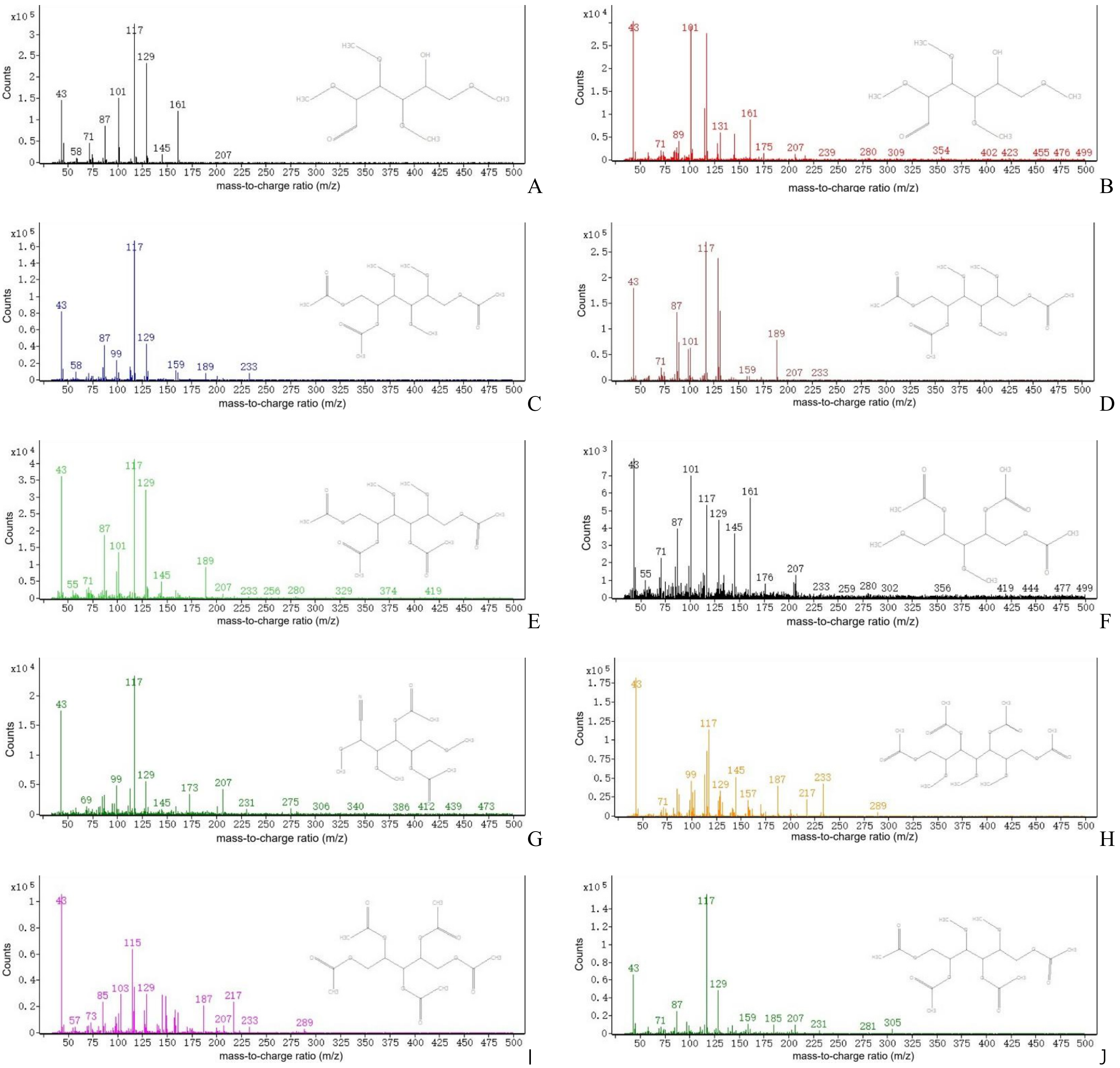


Figure S2. Partially O-methylalditol acetates formed on methylation analysis of PTPS-III.
